# Using the MDASI-Adolescent for Early Symptom Identification and Mitigation of Symptom Impact on Daily Living in Adolescent and Young Adult Stem Cell Transplant Patients

**DOI:** 10.3390/children9010019

**Published:** 2021-12-29

**Authors:** Irtiza N. Sheikh, Jeffrey Miller, Basirat Shoberu, Clark R. Andersen, Jian Wang, Loretta A. Williams, Kris M. Mahadeo, Rhonda Robert

**Affiliations:** 1 Department of Pediatrics, Pediatric Stem Cell Transplantation and Cellular Therapy, CARTOX Program, The University of Texas MD Anderson Cancer Center, Houston, TX 77030, USA; JAMiller1@mdanderson.org (J.M.); BAShoberu@mdanderson.org (B.S.); KMM@mdanderson.org (K.M.M.); 2 Department of Biostatistics, The University of Texas MD Anderson Cancer Center, Houston, TX 77030, USA; CRAndersen@mdanderson.org (C.R.A.); jianwang@mdanderson.org (J.W.); 3 Department of Symptom Research, Division of Internal Medicine, The University of Texas MD Anderson Cancer Center, Houston, TX 77030, USA; LoriWilliams@mdanderson.org; 4 Department of Pediatrics, The University of Texas MD Anderson Cancer Center, Houston, TX 77030, USA

**Keywords:** MDASI, patient-reported outcomes, stem cell transplant, pediatrics, adolescent/young adult, pain, quality of life, fatigue

## Abstract

Hematopoietic stem cell transplantation (HSCT) requires an intensive pre- and post-procedure course that leads to symptoms including fatigue, nausea/vomiting, and pain, all of which interfere significantly with activities of daily living. These symptoms place a substantial burden on patients during the time period surrounding transplant as well as during long-term recovery. The MD Anderson Symptom Inventory (MDASI) is a symptom-reporting survey that has been successfully used in adult patients with cancer and may have utility in the adolescent and young adult (AYA) population. At the Children’s Cancer Hospital at MD Anderson Cancer Center, we adopted a modified version of the MDASI, the MDASI-adolescent (MDASI-Adol), as a standard of care for clinical practice in assessing the symptom burden of patients in the peri-transplant period. We then conducted a retrospective chart review to describe the clinical utility of implementing this symptom-screening tool in AYA patients admitted to our pediatric stem cell transplant service. Here, we report our findings on the symptom burden experienced by pediatric and AYA patients undergoing stem cell transplantation as reported on the MDASI-Adol. Our study confirmed that the MDASI-Adol was able to identify a high symptom burden related to HSCT in the AYA population and that it can be used to guide symptom-specific interventions prior to transplant and during recovery. Implementing a standard symptom-screening survey proved informative to our clinical practice and could mitigate treatment complications and alleviate symptom burden.

## 1. Introduction

Hematopoietic stem cell transplantation (HSCT) in the pediatric and adolescent population requires an intense pre- and post-transplant treatment regimen. Patients receive chemotherapy as well as numerous other medications and can experience complications that affect their ability to perform activities of daily living [1]. In the period surrounding HSCT, patients often have symptoms such as painful mucositis, infection, fatigue, nausea/vomiting, decreased appetite, and psychological stress [2,3]. This symptom burden can lead to significant distress and reduce patients’ quality of life [1]. Studies describing the quality of life of long-term survivors of HSCT are few, although there is some evidence that patients’ quality of life improves over a period of months to years when compared to the immediate time period surrounding HSCT [4].

Common barriers to symptom control in patients receiving cancer-directed therapy include miscommunication between providers and patients, delays in interventions, and lack of age-appropriate patient-reported outcomes (PROs) [5]. Incorporating tools that assess patients’ symptoms into clinical practice can aid in overcoming these barriers by facilitating communication of symptoms between patients and providers [5,6]. Measurement tools that allow patients to report symptoms during or following cancer-directed care/HSCT may lead to earlier interventions to mitigate symptom severity [7]. Early symptom mitigation has been shown to lessen the distress that patients experience during treatment and improves their quality of life [8].

The MD Anderson Symptom Inventory (MDASI) is a 19-question PRO measure used to assess the symptom burden of 13 different symptoms and the extent to which the symptoms interfere with activities of daily life based on six domains: general activity, mood, work, relationships, walking, and enjoyment of life. The MDASI was developed by Cleeland et al. as a tool to measure the burden of the most frequent symptoms experienced by adult cancer patients [7]. The investigators discovered that while symptom scales such as the Symptom Distress Scale and the Memorial Symptom Assessment Scale (MSAS) are frequently used, they may not capture the extent of the symptoms experienced by patients and in the case of the MSAS, its length may be a hinderance for completion by patients [7]. The MDASI has been found to be reliable and validated in the adult (≥18 years old) cancer population and has been found to be reliable in adult patients receiving an autologous stem cell transplant [7,9]. The MDASI is successfully used in a variety of oncological fields and in international settings [10,11]. Similar to its counterpart in adult oncology, the adolescent MDASI, MDASI-Adol, is an easy-to-use tool based on the adult measure and assesses the severity of 13 symptoms at their worst in a 24 h period, including pain, nausea, and appetite loss, as well as the extent of the interference of the symptoms on patients’ daily life in the six domains [12]. The symptoms and symptom-related interferences elicited by the MDASI-Adol are described in Table 1. When compared to the MDASI used in the adult population, the interference item assessing “work (including work around the house)” was modified to “work (including school work and chores)” in the MDASI-Adol [7,12].

The MDASI-Adol was chosen as a symptom-reporting survey in the AYA population because it represents a PRO tool that allows communication between the clinician and patient and can be completed, and its results interpreted, in a relatively short time period. Moreover, the parallel adult measure currently used in adult oncology patients has been found to be reliable and validated in measuring the symptom burden of oncology patients, an aspect which may translate to the MDASI-Adol [7]. The bone marrow transplant (BMT) version of the MDASI has also been found to be internally reliable in assessing the symptom burden of adult patients undergoing autologous stem cell transplantation, another reason which contributed to the decision to use the MDASI-Adol in the AYA transplantation population [13].

On the MDASI-Adol, patients rate symptom severity at its worst in the last 24-h on a scale of 0–10 for each symptom, with 0 representing “not present” and 10 representing the symptom experience of “as bad as you can imagine.” Patients then rate the extent to which the symptoms interfere with daily living in the six domains including “general activity,” “mood,” “work (including school and chores),” ‘relations with other people,” “walking,” and “enjoyment of life.” Items measuring severity of interference are measured on a scale of 0–10, with 0 representing “did not interfere” and 10 representing “interfered completely.” The sum of the 13-symptom scores represent the “core” subscale score while the sum of the six interference scores designate the “interference” subscale score. In a study to measure adolescents’ perception of the meaning of the questions asked on the MDASI-Adol, forty-one patients between 13 and 17 years of age were cognitively debriefed on their understanding of the items of the MDASI-Adol [12]. Adolescents demonstrated an understanding of the symptom burden items; however, some comprehension difficulty was noted on the symptom interference items in younger adolescents (13- to 14-year-olds). Results of the cognitive debriefing led to minor changes in the wording of the interference items [12]. The MDASI-Adol is currently being psychometrically validated in patients 13–17 years of age.

Because early symptom reporting through surveys such as the MDASI has been important and effective, we utilized the adolescent-specific version of the MDASI (MDASI-Adol) in the adolescent and young adult (AYA) HSCT population to assess their symptom burden before and after HSCT and to improve PROs [7,12]. To most effectively use the MDASI-Adol for symptom reporting and assessment of resulting interventions, we established a standard of care practice to administer the MDASI to all patients admitted to the pediatric stem cell transplant unit at The University of Texas MD Anderson Cancer Center for HSCT-related care. The first phase of MDASI-Adol administration started in May 2020 at which time the MDASI-Adol was adopted by the inpatient HSCT team and administered to all patients admitted to the inpatient pediatric stem cell transplant service. The goal set by the team’s clinical administration was loosely defined as the implementation of a PRO tool provided to each patient during HSCT hospitalization to determine symptom burden around the time of transplantation. However, no standards were set for the timing of the initial survey administration to each patient, or the time intervals for repeated survey administrations. Therefore, patient survey completion was variable and somewhat provider dependent in this first phase. Beginning May 2021, the second phase was launched which outlined specific standards for the MDASI-Adol administration. Specifically, the MDASI-Adol was to be given to all patients admitted to the pediatric stem cell team within 3 days of admission (i.e., during the pre-transplantation conditioning phase of treatment), followed by weekly assessments throughout hospitalization and subsequent hospitalizations on the pediatric stem cell transplant service. In both phases, the MDASI-Adol was administered to adolescents and young adults 13 years of age or older who were admitted for HSCT or HSCT-related care to the pediatric stem cell transplant unit at MD Anderson Cancer Center. Patients younger than the age of 13, those unable to speak or comprehend English well enough to understand the questions on the survey, and patients with altered mental status were excluded from being administered the MDASI-Adol. The MDASI-Adol scores for each patient were collected on a paper-and-pencil form and the results were then uploaded to the electronic medical record (EMR). If a patient needed assistance, a staff member or caregiver/parent read the questionnaire verbatim (with no clarifications or additions) to the patient and recorded the answers. If the patient did not understand or did not answer, the item was left blank. MDASI-Adol results were entered into the patient chart in the EMR on the day on which it was completed.

Following completion of MDASI-Adol administration over a 14-month period, we conducted a retrospective chart review to assess the patient responses on the MDASI-Adol, compare the number and severity of symptoms between the two phases of administration (non-standardized vs. standardized), as well as assess the types and number of interventions and consultations performed by the medical team based on the responses on the MDASI-Adol. We also evaluated the change in symptom and symptom interference burden as reported on the MDASI-Adol in the time periods before and after transplantation. The primary aim of this retrospective review was to describe the clinical utility of using a brief symptom survey such as the MDASI-Adol in the AYA HSCT population, to describe the breadth of symptoms and the symptom interferences experienced by patients in the peri-transplant period as described by the tool, and determine the interventions undertaken based on the patient responses to the survey.

## 2. Methods

We conducted a retrospective study of all patients undergoing HSCT for either malignant or non-malignant diseases from May 2020 through July 2021, the time period when the MDASI-Adol screening was implemented as a quality improvement initiative to improve standardized symptom assessment among pediatric HSCT patients admitted to our pediatric stem cell unit. Patients included in this review were those that completed the MDASI-Adol at least 1 time before and/or after HSCT. Through extraction of data from the patients’ charts, we evaluated the symptom experience of AYA patients undergoing stem cell transplantation as captured by the MDASI-Adol and the clinical responses, or interventions, taken by the various medical staff members due to the patients’ MDASI-Adol. This study was approved by the Institutional Review Board at MD Anderson Cancer Center and due to the retrospective nature of the study, informed consent was waived.

Using the database provided by the stem cell transplantation team of patients admitted to the transplantation service from May 2020 to July 2021, and EMR review, we determined the number of patients eligible to complete an MDASI (>13 years old and able to comprehend English). By comparing this number with the actual number of patients that completed the MDASI-Adol, we were able to calculate the proportion of MDASI-Adol eligible patients who completed a survey before and/or after transplantation in both phases that were studied in this retrospective review. For each patient that met the inclusion criteria and completed the MDASI-Adol, the MDASI-Adol symptom subscale scores and interference subscale scores were totaled, respectively. In order to assess the interventions that occurred based on the results of the MDASI-Adol, we used the EMR to determine the number of times interventions and consultations were performed for each patient within one day of each MDASI-Adol completion. The types of consultation and interventions taken into account are described in Table 2. Using the day of stem cell transplantation as day 0, we also calculated the average days before and after transplantation that an MDASI-Adol was completed by a patient in both phases and we evaluated the number of surveys completed per patient, the number of symptoms reported per patient, and the number of interventions/consultations per patient in the two phases of MDASI-Adol administration.

In order to compare the burden of symptoms and the symptom-related interferences experienced by patients before and after transplantation, we also compared the symptom and interference scores, including the subscale scores, in the pre- and post-transplantation period. We also analyzed the number of patients and proportion of patients experiencing each symptom and symptom-related interference before and after transplantation in order to determine the prevalence of each symptom and interference within those time periods, respectively.

In order to compare scores before and after transplantation, scores from the pre-transplant time point utilized all available scores, while scores from the post-transplant time point utilized survey results within 90 days post-transplant, which are most relevant to the transplantation. Mixed-effect analysis of variance models were used to assess the association between each score and time point (pre-transplant versus post-transplant), blocking on patient to control for repeated measures. A 95% level of statistical significance was assumed in all statistical testing. Mixed-effect analyses were performed using R statistical software (R Core Team, 2020, version 3.6.3) [14]. Catseye plots were produced using the “catseyes” package [9,15].

## 3. Results

There were 30 patients admitted to the pediatric stem cell transplant service at MD Anderson Cancer Center for HSCT-related care from May 2020 through July 2021. During that time, 24 (80%) patients were eligible to complete the MDASI-Adol (≥13 years old and admitted to the pediatric stem cell transplant service before, during, or after transplantation). Of the 24 patients, 17 completed the MDASI-Adol in the first phase, prior to standardization of MDASI-Adol administration; 4 completed the MDASI-Adol in the second phase in which the MDASI-Adol was administered in a standard fashion (within three days of admission to the pediatric stem cell transplant service followed by weekly assessments); 2 patients declined to complete the MDASI-Adol at any point during their hospitalization; 1 patient was unable to complete the MDASI-Adol due to persistent altered mental status (AMS). Overall, three patients (13%) were unable to complete an MDASI-Adol at some time point during their admission owing to AMS, however, two of these patients were able to return to completing an MDASI-Adol following resolution of the AMS. The demographics of the patients surveyed are reflected in Table 3.

The median time of MDASI-Adol administration in phase 1, prior to standardized administration, was post-transplantation day + 17, with a range from day − 7 before transplantation to day + 842 after transplantation. Pre-transplantation surveys were administered at a median time of 3 days before the transplant (day − 3), and post-transplantation surveys were administered at a median time of day + 21. Of the 40 surveys administered, 33 (83%) were completed after transplantation (day + 1 or beyond). The median number of surveys completed per patient was 2 (range: 1–5). These characteristics of survey administration in both phases are described in Table 4. On MDASI-Adol surveys administered in the first phase, patients reported a mean of 14 symptoms and types of symptom-related interference on daily living (range: 3–19 per patient).

Next, subsequent symptom-related consultations and interventions were reviewed. For patients in the first phase, the total number of consultations and interventions occurring within 1 day after administration of the MDASI-Adol was 80 (mean: 5 per patient; range: 0–18 per patient). The most frequent interventions were (in order of frequency):

1. Evaluation by physical therapy and/or occupational therapy services;
2. The start of a patient-controlled analgesia opioid pump, initiation of opioids, initiation of pain medication for the relief of neuropathic pain, or an increase in the dose of pain medication including opioids;
3. Evaluation by a dietitian, including the start of total parenteral nutrition (TPN) or evaluation by the supportive care/palliative care staff for psychosocial support (these interventions occurred an equal number of times);
4. Evaluation by psychology or psychiatry staff and initiation or adjustment of psychotropic medication;
5. Evaluation by ancillary services such as child life services or art and music therapy;
6. Initiation or adjustment of antiemetic medication;
7. Initiation or adjustment of pharmacologic sleep aids.

In the second phase of MDASI-Adol implementation, 4 of 5 (80%) eligible patients were administered MDASI-Adol questionnaires before and after their stem cell transplants. One patient was unable to complete the MDASI-Adol at all owing to altered mental status. The timing of the surveys in this cohort complied with our conditions for using the MDASI-Adol as a standard of care symptom-assessment survey, i.e., patients were surveyed weekly (±3 days) following admission while inpatient. The median time of completion of the MDASI-Adol was day + 1, with a range from day − 72 to day + 218. Eight of the 15 (53%) completed surveys were administered after transplantation. The median number of surveys completed per patient was 3.5 (range: 1–7). Before transplantation, surveys were administered at a median time of day − 6, while post-transplantation surveys were administered at a median time of day + 12. Patients endorsed a mean of 17 symptoms and types of symptom-related interference with daily living (range: 16–19 per patient).

Subsequent symptom-related consultations and interventions were reviewed. The total number of consultations and interventions occurring within 1 day of administering the MDASI-Adol was 21 (mean: 7 per patient; range: 4–7 per patient). The most frequent interventions were (in order of frequency):

1. Evaluation by physical therapy and/or occupational therapy services;
2. Start of a patient-controlled analgesia opioid pump, initiation of as-needed opioid medication, initiation of pain medication for the relief of neuropathic pain, increase in the dose of pain medication including opioids;
3. Evaluation by ancillary services such as child life services or art and music therapy;
4. Initiation or adjustment of antiemetic medication;
5. Evaluation by the supportive care/palliative care team for psychosocial support, evaluation by psychology or psychiatry staff, and/or initiation or adjustment of psychotropic medication; or evaluation by a dietitian, including the start of TPN (these interventions occurred an equal number of times).

The differences in symptom burden before and after HSCT were also assessed. This was first accomplished by comparing MDASI-Adol scores before and after HSCT. The results of this comparison are summarized in Table 5. When evaluating MDASI-Adol scores of individual symptoms, lack of appetite increased the greatest by 2.2 units (*p* = 0.043) after transplantation followed by feeling drowsy increasing by 1.9 units (*p* = 0.07, a non-significant trend), and pain and fatigue both increased by 1.8 units (*p* = 0.12, 0.049, respectively). Overall, the total core subscale on the MDASI-Adol increased significantly after transplantation by 16.8 units (*p* = 0.047). Of note, all symptoms increased in severity following transplantation (range: 0.5–2.2 units) indicating that symptom burden increases following transplantation, even if marginally in some cases.

**Figure 1:**
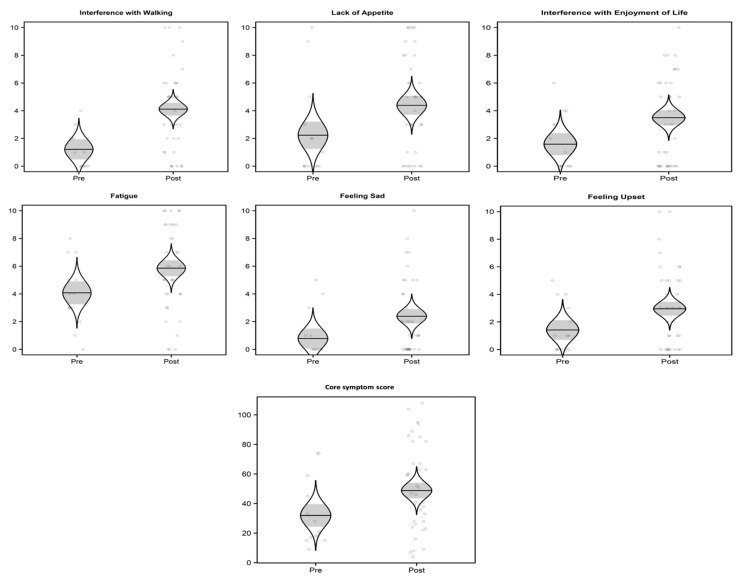
Summary of scores for each statistically significant symptom and symptom-related interferences before (“pre”) and after (“post”) the stem cell transplant. Scatterplots are overlaid by catseye plots illustrating the model-adjusted normal distribution of the means, with shaded ± standard error intervals. Scatterplots are randomly jittered slightly horizontally for clarity.

In terms of symptom-related interferences, interference with walking increased the greatest by 2.9 units (*p* = 0.002) following transplantation and was followed by an interference with enjoyment of life which increased significantly by 1.9 units (*p* = 0.041) following transplant. The total symptom interference subscale score also increased by 7.6 units (*p* = 0.07, a non-significant trend) following transplantation. Similar to the individual symptoms, the scores for each symptom interference item increased following transplantation (range: 0.3 to 2.9 units). Figure 1 illustrates the distribution of scores and comparison of mean scores pre- and post-transplant of the symptoms with statistical significance.

The next component of assessing the symptom burden before and after transplantation included comparing the prevalence of the symptoms between each time period. The three most common symptoms reported before and after transplantation were the same and included fatigue (median time of initial presentation: day − 5, range: day − 10 to + 10), disturbed sleep (median time of initial presentation: day + 3, range: day − 1 to + 17), and feeling drowsy (median time of initial presentation: day + 3, range: − 10 to + 16). With respect to all symptoms except numbness or tingling and difficulty remembering things, more than half of all patients experienced each symptom prior to transplantation (range: 44–89% of all patients). Moreover, following transplantation, more than half of all patients experienced each symptom except vomiting (range: 40–95% of all patients). Following transplantation, the largest increase following transplantation in the percentage of patients experiencing a specific symptom was dry mouth followed by feeling sad and pain.

In terms of symptom interference on daily living, interference with work, relationships, general activity, and walking were the most common prior to transplant. In comparison, following transplant, interference with general activity, mood, enjoyment of life, and walking were the most common sequelae of the symptom burden. Moreover, prior to transplantation, more than half of all patients (range: 56–86% of patients) experienced each of the symptom-related interference items, whereas following transplantation, more than 60% of all patients experienced each of the symptom-related interference items (range: 68–95% of patients). For each symptom-related interference item, there was also an increase in the proportion of patients following transplantation except in interference with work and interference with relationships. The largest increase in the proportion of patients experiencing a symptom-related interference was in interference with mood (39 units) followed by interference with enjoyment of life (28 units) and interference with general activity (17 units). Table 6 details the percentages of patients who experienced each symptom and symptom-related interference before and after transplantation.

## 4. Discussion

The aim of this project was to determine the utility of the MDASI-Adol in the AYA HSCT population to describe the symptom burden and the resulting symptom interferences experienced by patients in the peri-transplant period as well to describe the interventions/consultations performed due to the data gathered from the MDASI-Adol.

The use of the survey was implemented in two phases, with the second phase calling for MDASI-Adol administration within three days of HSCT-related hospital admission followed by rescreening on a weekly basis. In both phases, patients experienced multiple symptoms and symptom-related interferences, which resulted in appropriate interventions within 24 h of MDASI completion. Physical rehabilitation such as physical or occupational therapy was the most common intervention in both groups. This was followed by pain control measures as the second most frequent intervention, an observation supported by literature that pain experienced during HSCT can be complex and requires significant attention [16,17].

We also compared the MDASI-Adol scores and the prevalence of each symptom before and after transplantation to assess the difference in symptom burden between the two time periods. With respect to the symptom scoring, scores for lack of appetite, fatigue, feeling sad, and feeling upset increased the most in a statistically significant manner following transplantation. For symptom related interferences, scores for interference with walking and interference with enjoyment of life exhibited the greatest increase in a statistically significant manner on the MDAS-Adol following transplantation. The increase in severity in interference with walking may have contributed to physical rehabilitation as the most common intervention utilized. Keeping in mind that difficulty ambulating is an impediment to recovery following HSCT, there is an understanding in the HSCT community for the need to address the lack of standardized recommendations for physical therapy in the HSCT population [18]. Moreover, the increase after transplantation of MDASI-Adol scores in interference with walking and lack of appetite in our AYA population is similar to adult transplant recipients who report lack of appetite as one of the most severe symptoms and interference with walking as one of the most severe symptom-related interferences after transplantation [13,19]. Recognizing a lack of appetite is also important as decreased nutritional intake can have a deleterious effect on recovery following HSCT and may lead to complications and inability to tolerate certain interventions [20]. While there are no standardized guidelines in the AYA population for modes of nutrition delivery for those with difficulty tolerating oral intake during HSCT, early initiation of enteral nutrition or total parenteral nutrition (TPN) should be considered in patients unable to tolerate oral intake and/or at risk of malnourishment [20,21]. The increase in the MDASI-Adol score for fatigue following transplantation is consistent with the literature that indicates adolescents, especially those between 15–18 years old, were more likely to experience fatigue that is bothersome when compared to children [22]. The congruence of the MDASI-Adol fatigue scores with the literature may indicate that the MDASI-Adol has utility in accurately describing the increase in fatigue in the AYA population following transplantation considering that adult patients also report fatigue on the MDASI as one of the most severe symptoms experienced following HSCT [13,19].

As a symptom that increased by nearly two units following transplantation, feeling drowsy may have clinical significance when evaluating individual patients. This is especially important as adult patients frequently rate feeling drowsy on the MDASI as one of the most severe symptoms experienced throughout transplantation and further investigation of this phenomenon is needed in the AYA population. This may be achieved by sampling a larger patient population to assess the severity of drowsiness using the MDASI-Adol [13,19,22].

When reviewing the psychological components of HSCT and the observation that MDASI-Adol scores in feeling sad, feeling upset, and interference in enjoyment of life increased significantly following transplantation, there is a need for practitioners to acknowledge that HSCT can be associated with sadness, anxiety and depression in the immediate and long-term recovery period, in some cases far as six months to three years following transplantation [23]. This is also important considering that following transplantation, interference with mood and enjoyment of life were also one of the most common symptom-related interferences reported in our patient population, indicating that the increase in feeling sadness and feeling upset after transplantation may have an impact on the ability of patients to enjoy life in the peri-transplant period.

Moreover, the most common symptoms identified on the MDASI-Adol before and after transplantation were fatigue, disturbed sleep, and feeling drowsy. These results are similar to MDASI results in an adult autologous HSCT population where fatigue was one of the most common symptoms experienced at baseline as well as following transplantation, especially during the nadir, or the point after transplantation at which the white blood cell count is lowest [13]. In terms of disturbed sleep, while 95% of patients in our study endorsed sleep disturbance following transplantation, a significant percentage of adult patients also described difficulty sleeping as moderate or severe during the nadir [13]. In contrast, disturbed sleep is not a common symptom observed at baseline in the adult HSCT population assessed by the MDASI prior to transplantation [13]. Sleep disturbance in the adolescent population as one of the most common symptoms surrounding HSCT is important to recognize considering that disturbed sleep has been found to increase significantly during hospitalization for HSCT, especially during count recovery, and generally involves multiple etiologies [24].

While fatigue, disturbed sleep, and drowsiness were the most common symptoms prior to transplantation, it should also be noted that greater than 40% of patients experienced each symptom prior to transplantation and in some cases, the proportion of patients experiencing a specific symptom was greater than 75%. Moreover, greater than 50% of patients suffered from interference in daily living due to the symptoms. This indicates that at baseline, or prior to transplantation, a significant number of patients are experiencing difficulty in symptom management in both physical and psychological aspects. Moreover, as indicated by the proportion of patients experiencing the various interference items, a large number of patients are affected by these symptoms in their day-to-day living prior to transplantation. While there are studies that evaluate the impact of the symptom burden following transplantation on health-related quality of life (HRQoL) in the pediatric population, there is a significant lack of literature that explores the impact of these symptoms on HRQoL in the time period immediately prior to transplantation [25,26,27]. In contrast, adult studies have used the MDASI with success in order to quantify the symptom burden at baseline such as prior to autologous transplantation [13]. The importance of recognizing that pediatric and AYA patients may have a high symptom burden prior to transplantation is underscored by the fact that these patients may require support from the palliative care team upon admission or before preconditioning chemotherapy for transplantation has commenced [28]. Interdisciplinary patient care involving the stem cell transplant and palliative care teams has shown to improve the quality of life due to symptom burden mitigation in adult oncology patients and may have a role in the pediatric and adolescent population as well [29,30]. Early interventions such as physical activity before transplantation may translate to better outcomes such as decreasing fatigue, increasing QOL, and earlier discharge following HSCT compared to those not enrolled in a physical therapy program prior to HSCT [31]. In contrast, poor nutrition prior to HSCT may lead to delayed recovery and discharge from the hospital [32]. In summary, patients may require services as physical/occupational therapy, evaluation by a dietitian, evaluation by psychology/psychiatry much sooner than the time period following transplantation in order to optimize their physical and psychosocial health prior to undergoing HSCT as well as increase the likelihood of positive outcomes following transplantation.

Evaluating the symptom burden of AYA patients who have undergone HSCT is vital to understanding their unmet psychological and physical concerns [33]. Our use of the MDASI-Adol is in agreement with studies evaluating the MDASI in adult patients in that it confirmed an increase in the number of reported symptoms following transplantation. Moreover, patients in the AYA population were able to utilize the MDASI-Adol to identify those symptoms and communicate their severity on a scale of 0–10 [7,13,19,34,35]. Along with the significant increase in the MDASI-Adol score for the total core symptom subscale score as well as the increase in the total symptom-related interference subscale score following transplantation, these observations indicate that the MDASI-Adol is a tool that can be used to follow the evolution in the severity of specific symptoms and their interferences during an admission for HSCT. Our results demonstrate that following transplantation, physical limitations such as fatigue and difficulty ambulating and mental health issues and emotions such as sadness may interfere with a patient’s ability to adequately perform activities of daily living [36]. They may also interfere with a patient’s ability to enjoy life and perform general activities, two of the most common symptom-related interferences experienced by patients following transplantation in this study. Overall, the observations presented above and combined with the increase in the number of AYA patients experiencing most of the symptoms after transplantation agrees with the literature that HSCT is associated with a high symptom burden that spans physical and psychological manifestations [1,2,3]. The importance of early recognition of symptoms is underlined by the potentially negative impact on quality of life, especially in the early stages of recovery [1].

During the MDASI-Adol administration, we identified multiple points in the use of the survey that may benefit from changes and are supported by the literature on the use of PROs. We recognized further need for implementation planning and revised procedures in order to improve the utility of the MDASI-Adol in the AYA population. Here, we outline these observations and propose some possible clinical practice modifications.

We identified a need to develop a best practice standard for evaluating symptoms reported on the MDASI-Adol in patients with altered mental status. This is an instance where proxy reporting may serve as an alternative, considering that MDASI proxy reporting in adult brain tumor patients showed agreement with the patient’s symptom experience based on congruent scores between proxy and patient reporting [37,38,39]. This is especially important because altered mental status or encephalopathy can occur for many reasons after HSCT and is associated with dismal outcomes [40].

Historically, documentation in the medical record of patient-reported symptoms and interventions to relieve those symptoms, including in pediatric cancer, has been poor [41,42]. Thus, our team adopted standards for timely review of MDASI-Adol results and proper documentation of the scores and related interventions/consultations in the EMR. This standardized procedure for documentation of MDASI-Adol results on the day of MDASI-Adol submission by the patient served to ensure that the survey results were promptly reviewed, and that appropriate symptom-specific interventions would occur without delay. This effective use of the EMR to document and review the PRO measures could assist in early symptom mitigation [5,43]. Lack of effective documentation may lead to a lack of interventions for even the severest of symptoms, and even when interventions are initiated, they may not correlate with the symptoms reported on surveys [41]. In addition, timely review of the MDASI-Adol results allowed the stem cell transplant team to determine if worsening or improvement of symptoms correlated with the initiation of interventions or consultations.

Taking into account that not all eligible patients completed the MDASI-Adol, strategies that incentivize and increase the participation of patients are also needed. As reported for other pediatric specialties, strategies such as electronic symptom reporting or a point system may improve patient participation [5,44]. Exchange of feedback on symptom control between patients and providers during daily rounds and increasing awareness of the MDASI-Adol amongst the pediatric stem cell team may also help to increase patient participation rates [5,45].

Moreover, the patients included in this study were composed of diverse conditions, including malignant and non-malignant diseases. All patients in the malignant group, comprising 90% of the patients in this study, had past exposure to chemotherapy, which itself can predispose patients to short- and long-term adverse effects on quality of life [46,47]. This may partially explain the prevalence of symptoms and related interferences in patients prior to HSCT. The difference in symptom severity and its role in daily living may also be influenced by the directs effects of the various disease or tumor on a patient.

A limitation of the survey itself is that the MDASI-Adol is not a validated scale at this time and is currently undergoing further evaluations to analyze its psychometric properties for validation and internal reliability. Our reliance on the utility of the MDASI-Adol to recognize and quantify the symptom burden and interference on a 0–10 scale is based on the fact that the scale is similar to the one used in adult oncology which has been validated and found to be reliable [7]. Moreover, the MDASI-Adol is unable to screen patients <13 years old as younger adolescents may find it difficult to comprehend certain items assessed on the scale, especially the interference items [12]. This led to 5 of 30 (17%) patients ineligible to complete an MDASI-Adol due to their age (<13 years). The limitations of the use of the MDASI-Adol in the pediatric population as well as in those with altered mental status were two contributing factors to a small sample size of 21 patients. Due to the small sample size, it is possible that we may have not seen differences in severity that truly exist in certain symptoms and symptom-related interferences before and after transplantation. Conversely, the small sample size may have also introduced false-positive results. Therefore, our findings should be viewed as preliminary and exploratory. Future independent studies with larger sample sizes will be necessary to not only validate the findings but also to identify any possible additional factors.

Moreover, assessment time points varied across both groups due to the first phase of the MDASI-Adol administration. This limitation precluded further data exploration, including the ability to directly compare both groups. Moreover, in the absence of a control group which did not receive a transplant, reported change due to transplant is potentially confounded with change due to time or other unmeasured factors that influence symptom experience and their role in interfering with daily activities. Another limitation of our study is the reliance on input into the EMR in regard to the MDASI-Adol scores as well as interventions. Recovering data from the EMR in order to conduct a retrospective chart review presents challenges such as incomplete documentation or missing data. Moreover, our study was a retrospective chart review which relies on input of data into the patients’ chart from a third party, limits the ability to select a control group, as well as limits the ability to expand the diversity of the patient population in terms of age, gender, and disease [48]. Finally, statistical modeling assessed pre-to-post transplantation changes in 21 independent tests, without adjustment for multiple testing, so the results may include approximately one falsely significant difference (given the 95% level of statistical significance), however it is reassuring that the pre-to-post trends are consistent in direction regardless of significance.

Considering that the number of different symptoms may influence the variety of interventions required by a patient, larger retrospective and prospective studies may determine if the number of interventions truly correlate with the number of symptoms endorsed on the MDASI-Adol. This may assist in comparing the extent to which symptom-specific interventions impact the interference of those symptoms on daily living as well on HRQoL quality of life before and after HSCT. Moreover, studies with a larger number of patients may also identify more symptoms and related interferences on daily life that have significant differences in severity before and after transplantation due to the power that comes with a larger sample size [49]. Larger studies with reproducible results may find similar data as our study or work to further clarify the impact of HSCT on patients in terms of symptom severity and the burden on activities of daily living before and after transplantation. We are also interested in whether addressing symptom management via improved screening affects the rate of adverse events and/or the timeliness of hospital discharge. This evidence may be used to guide our intervention standards, which aim to achieve optimal symptom management and discharge timing. Further research is also needed to clarify the use of the MDASI-Adol in the outpatient setting and to compare the symptom burden and interventions to those in the inpatient setting. Future studies may also compare the symptom burden between AYA patients receiving various preconditioning regimens as well as those receiving autologous versus allogeneic HSCT. There is also a need to follow patients for months to years into their recovery from HSCT to determine the trajectory of the severity of their symptoms as well as the interventions required for relief. Moreover, while standardized administration of the MDASI in phase 2 resulted in more symptoms, symptom-related interferences, and interventions or consultations identified per patient, further studies are needed to determine the best method to administer the survey in terms of intervals between screenings and frequency of administration during hospitalization.

## 5. Conclusions

The use of a standardized symptom checklist for the early recognition of symptoms and their effects on daily living may be an effective tool to quantify symptom severity in AYA patients both before and after HSCT. Our data suggests that the MDASI-Adol is an effective tool in allowing patients to communicate the severity of symptoms before and after transplantation and the survey can be used to determine the impact of those symptoms in interfering with aspects of daily living such as walking, enjoying life, and relationships, amongst others that were identified on the MDASI-Adol. Use of the MDASI-Adol facilitated symptom reporting by adolescents and young adults and thereby established a form of communication between these patients and their clinicians. Management of symptoms during HSCT is important for improving patients’ quality of life while they prepare for a transplant, undergo the intensive procedure, and recover. Future studies are required to determine if the MDASI-Adol is validated and reliable in the adolescent population.

## Figures and Tables

**Table 1:** Symptoms and the domains of daily living in which those symptoms interfere as described on the MDASI-Adolescent (MDASI-Adol) survey.

Symptoms	Symptom Related-Interferences
Pain	General activity
Fatigue	Mood
Nausea	Work (including schoolwork and chores)
Disturbed sleep	Relationships
Feeling disturbed or upset	Walking
Shortness of breath	Enjoying life
Trouble remembering things	
Lack of appetite	
Feeling drowsy	
Dry mouth	
Feeling sad	
Vomiting	
Numbness or tingling	

**Table 2:** Types of interventions and consultations that occurred within one day of MDASI-Adolescent (MDAS-Adol) administration.

Consultations	Interventions
Physical and/or occupational therapy	Initiation of a patient-controlled analgesia opioid pump
Nutritional services (i.e., dietitian)	Initiation or adjustment of pain medication, including increasing doses of intravenous or oral opioids
Supportive/palliative care staff	Administration of pain medication intended to relieve neuropathic pain
Psychotherapy or psychiatry services	Increase in pain medication dosage, including that of opioids
Ancillary services (e.g., child life, art and music therapy)	Initiation or adjustment of psychotropic medication
	Initiation or adjustment of antiemetic medication
	Initiation or adjustment of pharmacologic sleep aids

**Table 3:** Demographic and clinical characteristics of patients who submitted an MDASI-Adolescent (MDAS-Adol) (*N* = 21).

Characteristic	No. of Patients ^a^
Age, median (range), y	20 (13–25)
Sex, *N* (%)	
Male	12 (57)
Female	9 (43)
Race/ethnicity, *N* (%)	
Hispanic	8 (38)
White	8 (38)
Black	3 (14)
Asian	2(10)
Reason for HSCT, *N* (%)	
Malignancies	
ALL	7(33)
AML	6 (28)
Mixed lineage leukemia	1(5)
Hodgkin lymphoma	3(14)
Myelodysplastic syndrome	1(5)
Medulloblastoma	1(5)
Nonmalignant conditions ^b^	2(10)

^a^ Unless otherwise indicated. ^b^ Includes aplastic anemia and sickle cell disease. Abbreviations: HSCT, hematopoietic stem cell transplantation; AML, acute myeloid leukemia; ALL, acute lymphocytic leukemia.

**Table 4:** Characteristics of MDASI-Adolescent (MDAS-Adol) surveys completed in both phases of administration.

	Phase 1	Phase 2
Number of patients	17	4
Time of MDASI-Adol administration, median (range), day of transplantation		
Overall	+17 (−7 to +842)	+1 (−72 to +218)
Pretransplantation	−3	−6
Posttransplantation	+21	+12
Surveys completed		
Overall, per patient, median(range)	2 (1–5)	(1–7)
Pre-transplantation (*n*,%)	7, 17	7, 47
Post-transplantation (*n*,%)	33, 83	8, 53
Number of reported symptoms and symptom related-interferences per patient, average (range)	14 (3–19)	17 (16–19)
Number of interventions and consultations, total, per patient (range per patient)	80, 5 (0–18)	21, 7 (4–7)

**Table 5:** Mixed-effect model summaries of change in scores following stem cell transplantation. Scores on the MDASI-Adolescent (MDASI-Adol) before (pre) and after (post) transplant were compared with the difference in scores detailed in the “estimate” column.

Symptoms	Contrast	Estimate	SE	*p*-value
Lack of Appetite	Post—Pre	2.2	1.0	**0.043**
Feeling Drowsy	Post—Pre	1.9	1.0	0.07
Pain	Post—Pre	1.8	1.1	0.12
Fatigue	Post—Pre	1.8	0.9	**0.049**
Dry Mouth	Post—Pre	1.6	1.1	0.16
Feeling Sad	Post—Pre	1.6	0.7	**0.031**
Feeling Upset	Post—Pre	1.5	0.7	**0.046**
Disturbed Sleep	Post—Pre	1.2	1.0	0.22
Shortness of Breath	Post—Pre	1.2	0.7	0.08
Numbness or Tingling	Post—Pre	1.0	0.7	0.15
Nausea	Post—Pre	0.6	1.0	0.53
Remembering Things	Post—Pre	0.5	0.9	0.59
Vomiting	Post—Pre	0.5	1.1	0.64
Symptom-Related Interferences				
Interference with Walking	Post—Pre	2.9	0.9	**0.002**
Interference with Enjoyment of Life	Post—Pre	1.9	0.9	**0.041**
Interference with General Activity	Post—Pre	1.4	1.0	0.17
Interference with Mood	Post—Pre	1.0	0.9	0.26
Interference with Work (School/Chores)	Post—Pre	0.4	0.9	0.64
Interference with Relations with Other People	Post—Pre	0.3	0.9	0.70
Subscale scores				
Core	Post—Pre	16.8	8.1	**0.047**
Interference	Post—Pre	7.6	4.1	0.07

Bold indicates *p*-values with statistical significance.

**Table 6:** Percentage of patients experiencing symptoms and symptom-related interferences before and after stem cell transplantation.

Symptoms	Patients with Symptoms Pre-Transplantation (%)	Patients with Symptoms Post-Transplantation (%)	Difference between Timepoints (%)
Fatigue	89	95	6
Disturbed sleep	89	95	6
Feeling drowsy	89	90	1
Lack of appetite	86	85	−1
Feeling upset	86	80	−6
Shortness of breath	83	85	2
Nausea	78	75	−3
Pain	67	85	18
Dry mouth	56	80	24
Feeling sad	56	75	19
Difficulty with remembering things	56	70	14
Numbness or tingling	44	55	11
Vomiting	44	40	−4
Symptom-Related Interferences			
Interference with work (school/chores)	86	74	−12
Interference with relations with other people	86	68	−18
Interference with general activity	78	95	17
Interference with walking	78	84	6
Interference with mood	56	95	39
Interference with enjoyment of life	56	84	28

## Data Availability

Data sharing is not applicable to this article.
